# Analysis of the Position Recognition of the Bucket Tip According to the Motion Measurement Method of Excavator Boom, Stick and Bucket

**DOI:** 10.3390/s20102881

**Published:** 2020-05-19

**Authors:** Dongik Sun, Changuk Ji, Sunghoon Jang, Sangkeun Lee, Joonkyu No, Changsoo Han, Jeakweon Han, Minsung Kang

**Affiliations:** 1 Department of Mechatronics Engineering, Hanyang University, 55 Hanyangdaehak-ro, Sangnok-gu, Ansan-si, Gyeonggi-do 15588, Korea; jeniussdi@hanyang.ac.kr (D.S.); dltkdrms@hanyang.ac.kr (S.L.); njkonly@hanyang.ac.kr (J.N.); 2 OHSUNG SYSTEM CO., LTD., 62 0 504ho, Gwangdeokseo-ro, Danwon-gu, Ansan-si, Gyeonggi-do 15461, Korea; jcw2074@naver.com; 3 ROHAU CO., Ltd., Hanyang University, 55 Hanyangdaehak-ro, Sangnok-gu, Ansan-si, Gyeonggi-do 15588, Korea; shjang@rohau.co.kr; 4 Department of Robot Engineering, Hanyang University, 55 Hanyangdaehak-ro, Sangnok-gu, Ansan-si, Gyeonggi-do 15588, Korea; cshan@hanyang.ac.kr; 5 Department of Smart Interdisciplinary Engineering, Hanyang University, 55 Hanyangdaehak-ro, Sangnok-gu, Ansan-si, Gyeonggi-do 15588, Korea

**Keywords:** excavator, bucket position recognition, excavator modeling, inertial measurement unit, cylinder length measurement with draw-wire sensor

## Abstract

On modern construction sites, guidance and automation systems are increasingly applied to excavators. Recently, studies have been actively conducted to compare the estimation results of the bucket tip with the motion measurement method of the boom, stick, and bucket and the sensor selection. This study selected the method of measuring the cylinder length of boom, stick, and bucket, and the method of directly measuring the boom, arm, and bucket, which are commonly used in guidance and automation systems. A low-cost sensor that can be attached and detached to the excavator in modular form was selected to apply the above methods to commercial excavator. After the sensor selection, hardware and excavator simulation models for sensor measurements were constructed. Finally, the trajectory of the bucket tip was compared and analyzed through graphs and simulation results when the boom, stick, and bucket were independently rotated one by one, or together. The results gives a guideline on what kinds of sensors would be better in machine guidance or controlling an excavator according to given external environments.

## 1. Introduction

Recently, construction sites can get higher efficiency by virtue of excavator having machine control, guidance, and/or additional systems made for expressing the environments. The most significant matter in the aforementioned techniques is the estimation of the end-point of the excavator precisely by using an appropriate sensor device. However, there are many difficulties in estimating the end-point of the excavator precisely through the sensor system because of inherent characteristics such as engine vibration, large gravity force, or unpredictable disturbance [1]. Thus, the performance of estimation is up to a selection of sensor devices measuring kinematic parameters, and it should should be considered according to the characteristics of the working site.

Methods for position estimation of the end-point of excavator are generally classified as follows: (1) measuring the length of cylinders; (2) doing the link motion for estimating the angle displacements indirectly; (3) knowing the angle displacements directly; and (4) considering the hydraulic system.

In the case of the cylinder length, the displacements of the cylinder can be measured by using an optional bracket and guide for attaching the linear encoder or stroke sensors. Then, parameters in the derived mathematical equation are substituted by output data of sensors so that the end-point can be estimated. In the case of the link motion, inertia measurement unit (IMU), tilt, and/or accelerometer sensors are used and attached to each link for measuring the joint angle indirectly through the rotation of the boom, stick, and bucket links. These measured data are also used for estimating the end-point of excavator through the derived kinematic modeling. In the case of the measurement of the revolute joints of the excavator, the rotary encoder, resolver, or potentiometer have been used for measuring the angular displacements of the joint directly. The last cases are related to the hydraulic flow rate. The flow rate sensors are inserted into the pipeline, and then devices measure the flow rate so that the angular displacement of each joint that is used for estimating the end-point of the excavator can be estimated [2].

The case study about the use of these sensors at excavators can be found easily. Many studies described the application for machine control, guidance, path planning, collision avoidance, etc. Table 1 showd the most recent studies about sensors application in the excavator. This table also includes additional sensors that are not mentioned above such as vision, real-time location system, etc. Examples of applications in cylinder length and link motion measurements form a large majority.

**Figure 1:**
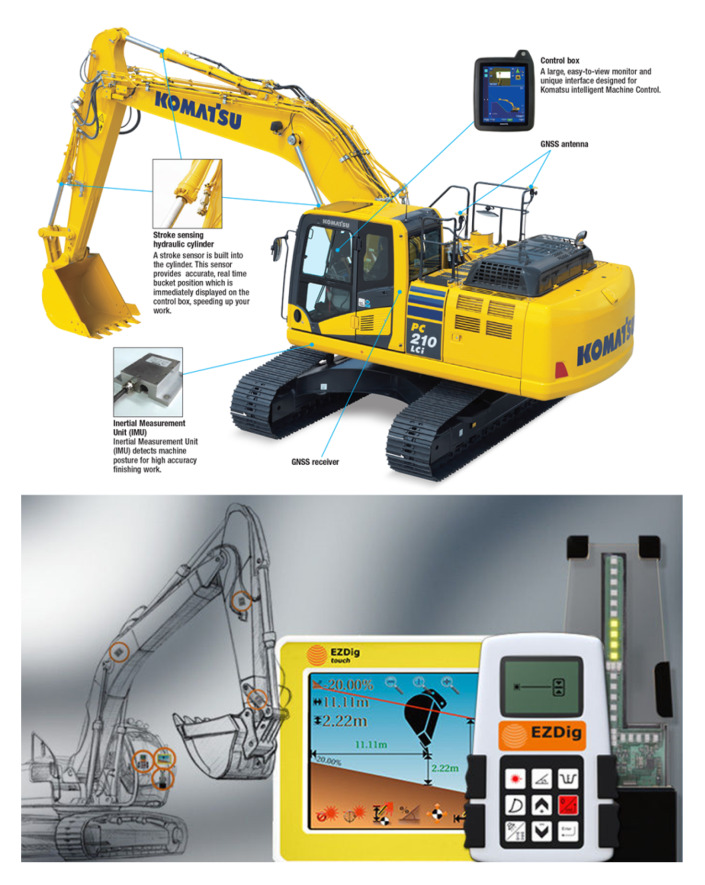
(**top**) Machine guidance using measured cylinder length, which is developed by KOMATSU; (**bottom**) Machine guidance using measured links motion, which is developed by EZDig.

A review on the sensor systems of the next generation construction machinery is present in the [39]. The cited papers under “Other” in Table 1 are about the development sensors. Figure 1 shows some representative examples of sensors application.

The third and fourth cases require some modifications and renovations to the mechanical system of excavator for measuring the desired parameters. Additionally, when it comes to the fourth cases that are related to the hydraulic system, there are many non-linearities so that complementary filtering jobs should be progressed for making clear signals [40,41]. In contrast, the first and second cases can reduce and minimize the modifications of the mechanisms of the excavator if additional brackets are used. These ways can preserve the original status of the excavator. Thus, the first two methods are commonly used in practical cases at construction sites since they are economical and do not damage the property (Table 1). Therefore, this paper deals with these two methods: the measurements of the cylinder length, the measurements of the link motion for measuring the angular displacements of each joint indirectly.

However, the aforementioned two ways are completely different with respect to the principal of end-point estimation. In the case of the measurement of cylinder length, each cylinder movement has no effect on the other output data because the principal about the motion of the cylinder comes from the absolutely independent mechanism in the joint space. Then, the end-point of the excavator can be estimated through forward kinematics and the derived formula which converts each cylinder length into the joint angle displacement. In contrast, measuring the motion of each link has an effect on the other output data. In general, tilt sensors or inertial measurement units (IMU) devices are attached to the links by using an additional bracket. Then, they measure the absolute angle displacements of each joint indirectly with respect to the fixed base frame. Consequently, each motion of link results in changing the other link position with respect to the view of the global frame. For this reason, some calibration works must be progressed since output data to be inputted into the forward kinematics formula must be relative angle displacement. Additionally, the location of sensor devices to be attached must be considered for making calibration easier.

These two methods have one common feature: renovations and modifications of the mechanism and hydraulic system are not required in the installation. This can also reduce the time and cost. Most importantly, these two methods are already widely adopted by many construction companies in order to help the user to make a rapid automation and guidance system without damaging their property.

Even though machine guidance and control systems have been developed by many construction companies, studies on the analysis of sensor characteristics regarding the application to excavators have been not public since these are confidential property. Moreover, high-cost devices are used in the commercial guidance system. In the case of papers cited in Table 1, almost all papers have adopted sensor systems depending on the experimental environment and circumstances. Some studies have adopted various types of sensor systems. This means that the effectiveness of each sensor in measuring excavators is different. Therefore, studies on improving the performance at a low cost are still need. In this situation, it is worth studying the sensor characteristics in estimating the end-point of excavator for giving a guideline on what type is better or worse according to the case [18,42].

As mentioned above, low-cost commercial sensor devices that are attached and detached to excavator links easily were selected to analyze the characteristics and suitability when they are applied for estimating the end-point. The reason low-cost devices were selected is that almost all of the developments begin with at a low cost. The analysis was progressed by comparing the estimated trajectory of the bucket made by logged sensor output data. The sensor brackets and communication systems for logging were constructed. The CAD model and simulation tool were also used for checking the validation.

Section 2 describes the kinematic modeling for estimating the end-point of the excavator. The system construction for sensors application ise explained in Section 3 and the experimental setup and results are described in Section 4. Lastly, a discussion of the results is presented in Section 5.

## 2. Kinematic Modeling of the Excavator

### 2.1. Forward Kinematics: Revolute Joint

**Figure 2:**
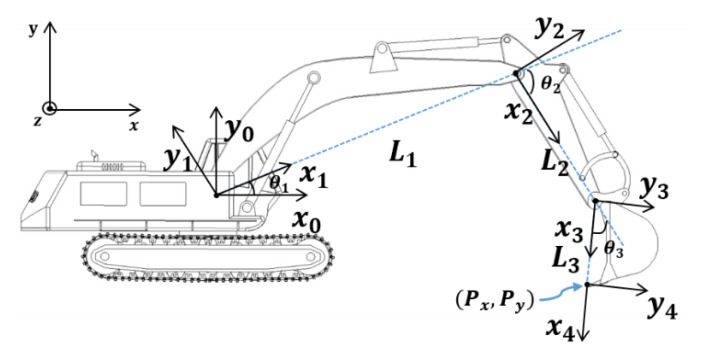
Definition of the axis and frame with respect to base when undercarriage of the excavator is fixed to the ground.

To estimate the end-point of the excavator using output sensor data, the derivation of the forward kinematics of this machine must be performed. A typical excavator has three degrees of freedom with respect to side view (two-dimensional space) if the swing of the cabin is excluded. Figure 2 shows the two-dimensional schematic and frame. The Denavit–Hartenberg table based on Figure 2 is shown in Table 2.

The end-point position of the excavator can be expressed as x and y values with respect to the defined base frame in Figure 2, and the results of forward kinematics are as follows.

(1) $$P_{x}=L_{1}c_{1}+L_{2}c_{12}+L_{3}c_{123}$$

(2) $$P_{y}=L_{1}s_{1}+L_{2}s_{12}+L_{3}s_{123}$$

### 2.2. Forward Kinematics: Cylinder Length

**Figure 3:**
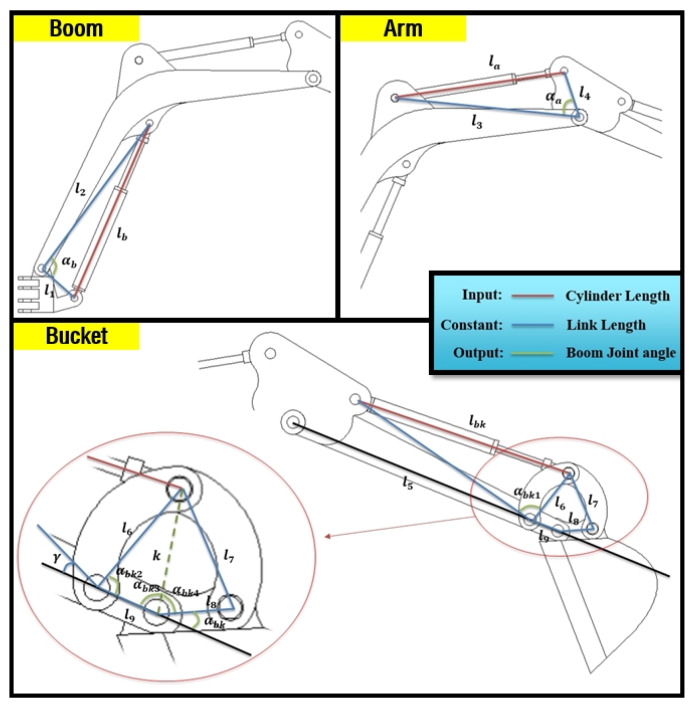
A simple schematic of the boom, stick, and bucket cylinder illustrating the specific angle and cylinder length.

In the above section, $\theta_{1}$, $\theta_{2}$, and $\theta_{3}$ are necessary elements in estimating the position of the end-point. Thus, cylinder length must be converted to $\theta_{1}$, $\theta_{2}$, and $\theta_{3}$ through the derived mathematical equations. The most important parameters in converting to revolute joint angle are $\alpha_{b}$, $\alpha_{a}$, and $\alpha_{bk}$, which are illustrated in Figure 3. $\alpha_{b}$ can be derived by Equation (3), $\alpha_{a}$ comes from Equation (4), and $\alpha_{bk}$ can be obtained using Equations (5)–(9).

(3) $$\alpha_{b}=cos^{-1}\left(\frac{{l_{1}}^{2}+{l_{2}}^{2}-{l_{b}}^{2}}{2l_{1}l_{2}}\right)$$

(4) $$\alpha_{a}=cos^{-1}\left(\frac{{l_{2}}^{2}+{l_{3}}^{2}-{l_{a}}^{2}}{2l_{2}l_{3}}\right)$$

(5) $$\alpha_{bk1}=cos^{-1}\left(\frac{{l_{5}}^{2}+{l_{6}}^{2}-{l_{bk}}^{2}}{2l_{5}l_{6}}\right)$$

(6) $$k=\sqrt{{l_{6}}^{2}+{l_{9}}^{2}-2l_{6}l_{9}cos\left(\alpha_{bk2}\right)}$$

(7) $$\alpha_{bk4}=cos^{-1}\left(\frac{{l_{8}}^{2}+k^{2}-{l_{7}}^{2}}{2l_{8}k}\right)$$

(8) $$\alpha_{bk3}=cos^{-1}\left(\frac{{l_{9}}^{2}+k^{2}-{l_{6}}^{2}}{2l_{9}k}\right)$$

(9) $$\alpha_{bk}=\pi -\alpha_{bk3}-\alpha_{bk4}$$

## 3. System Setup for Validation

### 3.1. Verification of the Derived Kinematic Equations

To evaluate the derived equations, this paper selected and analyzed the real physical excavator Vio-17 made by YANMAR.

**Figure 4:**
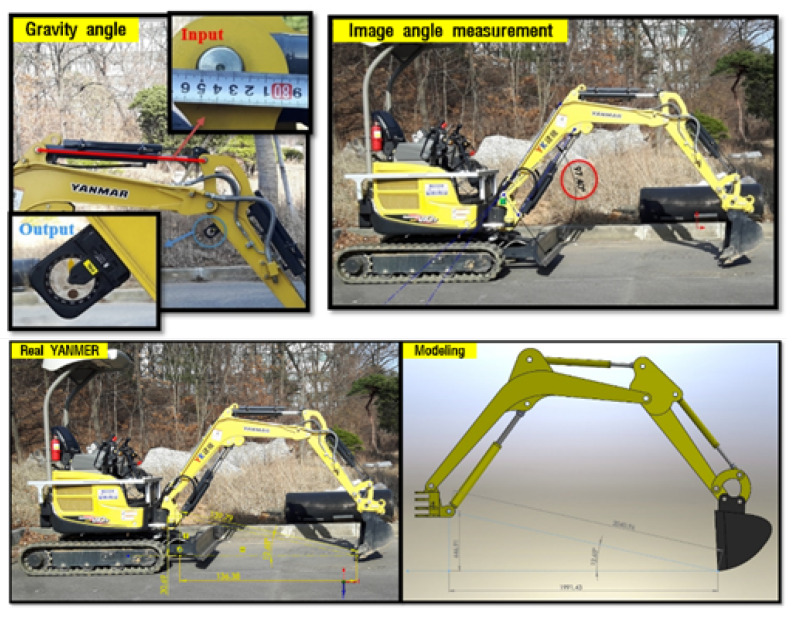
The illustration of the measurement methods: protractor and imagery.

The 3D CAD model was made for validation of the derived equations through the simulation of end-point estimation by inputting the sensors’ output data of cylinder length and joint angle from link motions. In fact, the specifications of Vio-17 were not provided as they are a confidential asset of the company, thus the CAD model was done through manual measurement. Therefore, to correct errors in manual measurements, the produced CAD model must be verified. First, real $\alpha_{b}$, $\alpha_{a}$, and $\alpha_{bk}$ were measured by using both the protractor and imagery (Figure 4). In the case of the image method, the excavator must be photographed from the side without inclination for making the two-dimensional space absolute. Then, the image was inserted into the CAD utility for measuring the desired value [43,44].

**Figure 5:**
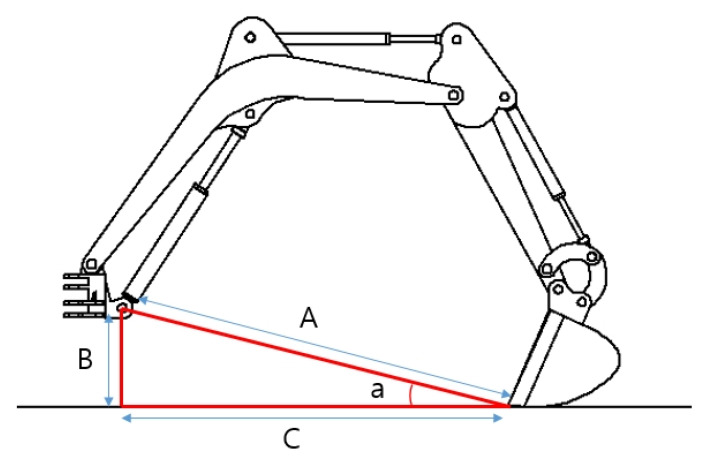
Reference for validation of the CAD model: (**A**) diagonal; (**B**) height; (**C**) length; and (**a**) the angle created by A and B.

By virtue of these two methods, derived equations were checked and the CAD model was also evaluated. Actual measurements gave the following result: the image method is better than the protractor. Thus, the result of the simulation for estimating the end-point of the excavator was compared by the result of the image method with respect to Figure 5. Table 3 is the result of the comparison between the simulation and image methods. This table gives validation of the CAD model and forward kinematics by showing an allowable error, 1.3 cm. In the construction field, if the error of the machine guidance is within 2 cm, it is enough to be used practically [17]. For this reason, this CAD model was used for analyzing the sensor characteristics in estimating the end-point of the excavator.

### 3.2. Construction of the Sensor System

The sensor system construction was divided into two parts, namely those regarding cylinder length and link motion, and both were progressed without modifications and renovation of the mechanical system.

**Figure 6:**
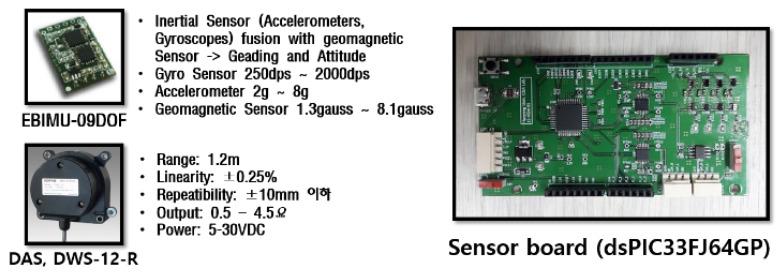
The Specifications of the draw-wire and IMU sensors.

**Figure 7:**
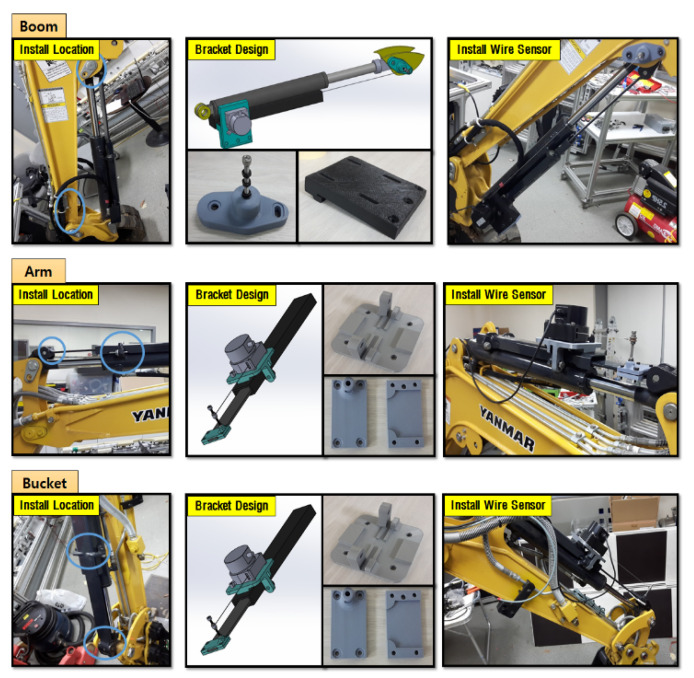
Developed brackets for draw-wire sensors and status of the installation.

**Figure 8:**
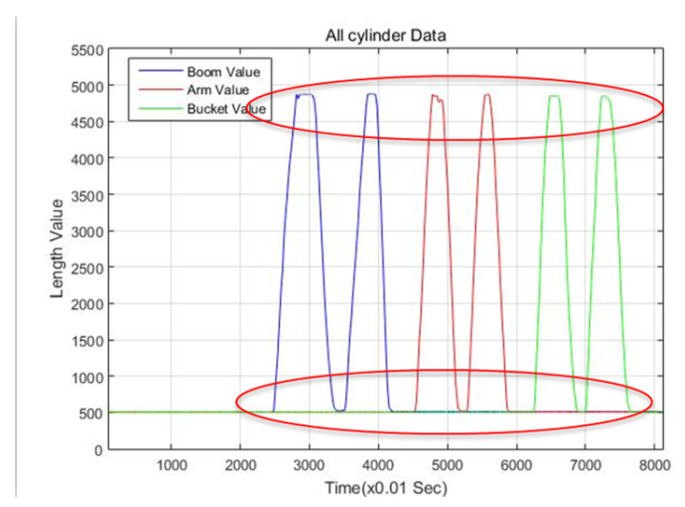
The graph showing the wire sensor’s output data of the maximum and minimum cylinder length related to each link.

First, to estimate the end-point of the excavator through the measurements of each cylinder length, a low-cost draw-wire sensor of linear potentiometer type that could be easily attached to the link was selected. The specifications of this selected device is shown in Figure 6. The price of one draw-wire sensor was about 150 USD. Thus, the total price of sensor devices for measuring the boom, stick, and bucket cylinder displacements was 450 USD. This is somewhat low cost because, in general, the encoder type of the draw-wire sensor is more expensive than the potentiometer type. Figure 7 shows the attached draw-wire sensors to each link parallel with customized brackets. In the case of boom link, the bracket for fixing the draw-wire sensor was attached near the boom joint, as shown at the top of Figure 7. Finally, in the case of the stick and bucket links, brackets were fixed to the cover of the cylinder so that all draw-wire sensors could measure the absolute length displacements of the cylinders. The transmission cycle of the sensor systems esd 10 ms. The embedded system for logging the data was also made. Figure 8 shows the minimum and maximum measurement output data of each cylinder length and their linearity.

**Figure 9:**
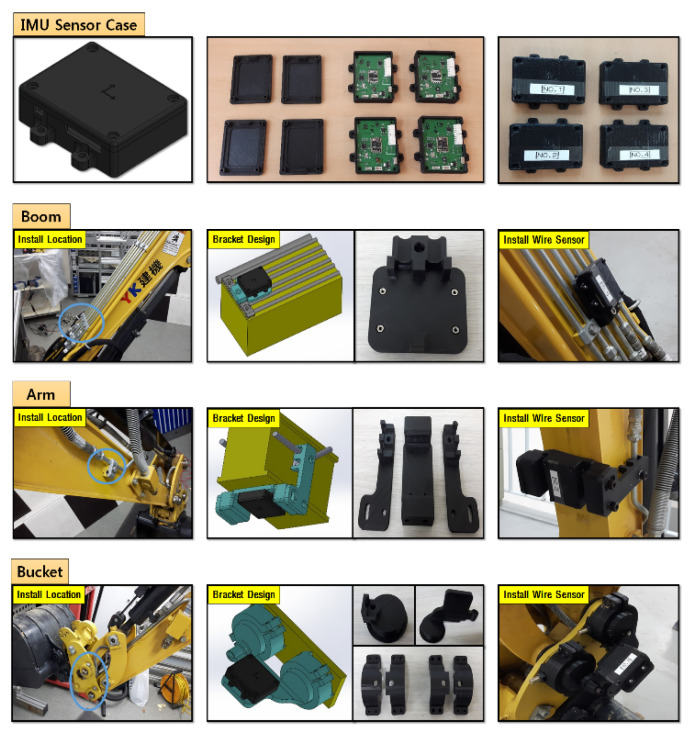
Developed brackets for IMU sensors and status of the installation.

**Figure 10:**
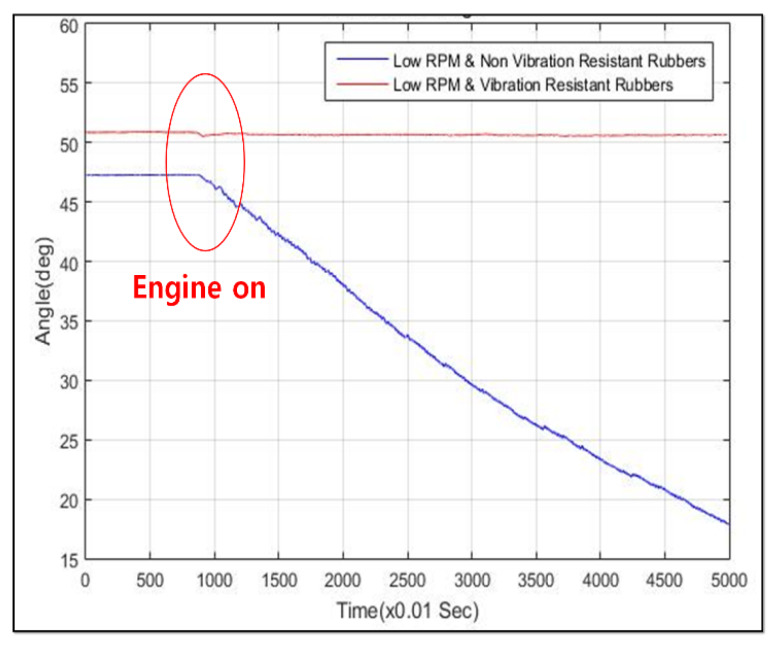
The importance of the anti-vibration system: drift occurred when anti-vibration rubber was not applied.

Second, to estimate the end-point through the measurements of each joint angle displacements indirectly by measuring the rotation of links motion, a low-cost IMU sensor device was also selected named EBIMU-09DOF. This device can transmit the raw data so that the inherent characteristics of the excavator itself can be analyzed, and its specifications are explained in Figure 6. The price of one IMU sensor was about 180 USD. Thus, the total price of sensor devices for measuring the boom, stick, and bucket cylinder displacements was 540 USD. This is a very low-cost device. The cost range of IMU devices is very large. For example, the attitude/heading reference system (ARHS) device made by machine guidance company averages over 1000 USD. Brackets were made and installed on each link, as shown in Figure 9. In the case of boom and stick links, the brackets for fixing the IMU sensors were attached to the boom and stick link, as shown at the top of Figure 9. Finally, in the case of the bucket link, the bracket was fixed to two revolute joints of the four-bar linkage so that all IMU sensors could measure the absolute angle displacements of joints indirectly through calibration. The reason calibration is needed in this process is that the slopes of brackets and links are not the same. The angle displacements can be measured correctly through the calibration. The transmission cycle of the sensor systems was 10 ms. When brackets were installed on the link, the butadiene rubber was used for anti-vibration, which prevents the data drift. Figure 10 illustrates drift generation for when rubber was and was not used. This device transmits three kinds of data: roll, pitch, yaw. In general, roll data are the most robust to disturbance and have the widest measurable range [45,46]. Thus, the roll data were chosen for measuring the joint angle.

## 4. Experiments and Results

**Figure 11:**
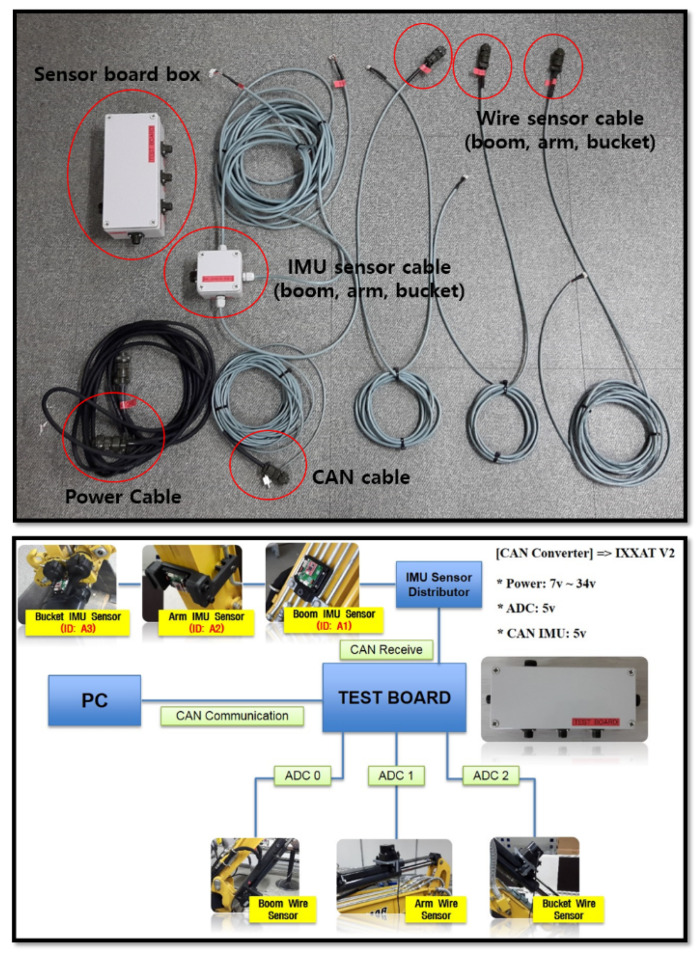
A schematic of the overall communication system for gathering the desired data.

The CAN (Controller Area Network) communication system was built in the embedded system for logging the output data of both draw-wire and IMU devices at the same time (Figure 11). These output data were inputted into the derived equations for estimating the end-point. To compare characteristics between two devices in the estimation of the end-point, Simulink of MATLAB was applied to these experiments.

**Figure 12:**
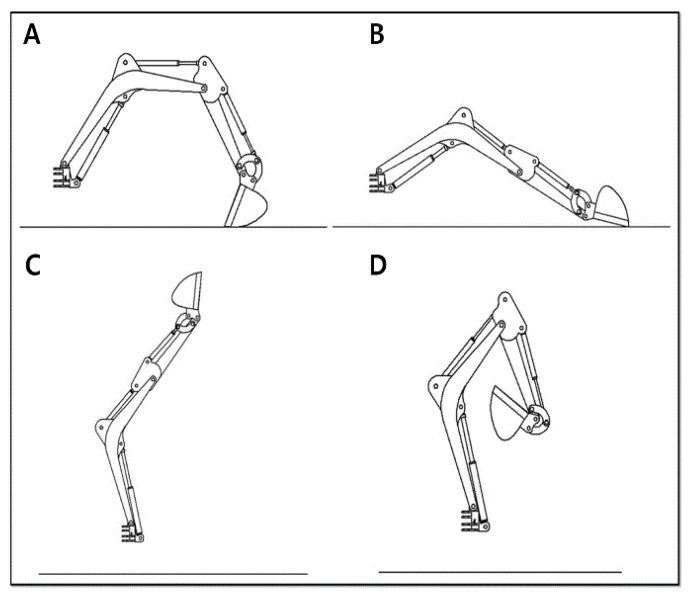
The four main poses of the excavator to synchronize the initial and final points by inserting the offset value. (**A**) the middle point in aspect of the horizontal side view; (**B**) the pose when all links of the excavator are stretched as much as possible; (**C**) the pose when the excavator making a maximum height; (**D**) when all cylinders have a minimum length.

**Figure 13:**
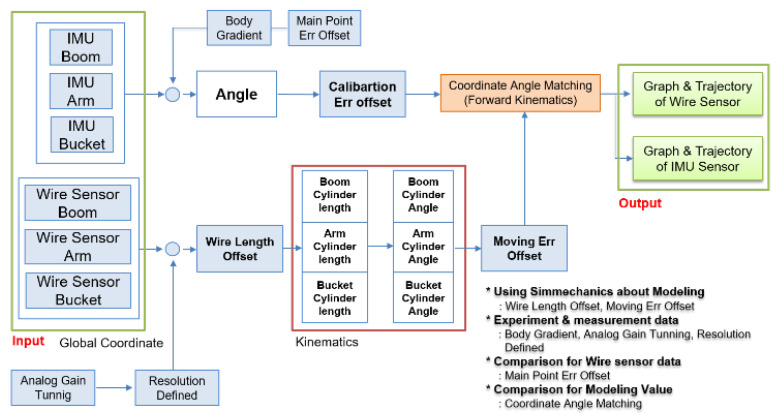
Flow chart of the phases of data collection from both sensors through the compensation process.

The four main poses of the excavator were chosen (Figure 12). Figure 12A is the middle point in the aspect of the horizontal side view. Figure 12B is the status when all links of the excavator are stretched as much as possible. Figure 12C is performed for making a maximum height. Figure 12D is when all cylinders have a minimum length. The IMU and draw-wire sensors measured the pose information for the poses in Figure 12A–D and then the logged data were calibrated to synchronize the initial and final points by inserting the offset value. This processed data were finally applied to the derived equations to estimate the end-point of the excavator. The entire process is shown schematically in Figure 13.

**Figure 14:**
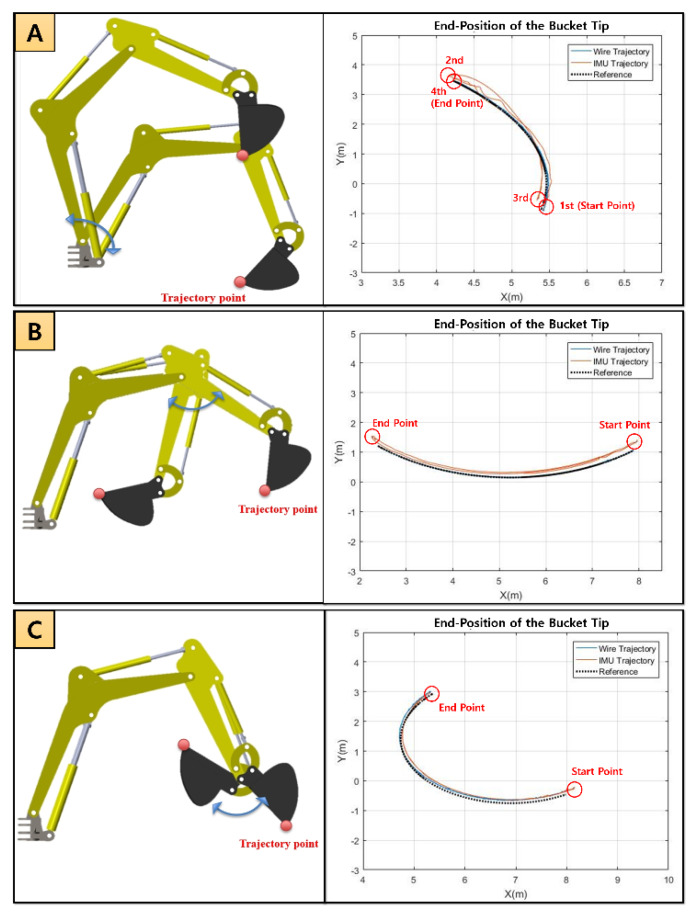
A comparison between the results of the collected data and the reference bucket trajectories. (**A**) Only boom motion; (**B**) Only stick motion; (**C**) Only bucket motion.

The characteristics of bucket tip were analyzed by measured data from draw-wire and IMU sensor during the motion of the boom, stick, and bucket, separately. In the analysis of the bucket tip, the non-motion part of joints was fixed for measuring the rotating part, and the reference trajectory was generated through the simulation created using Simulink. The length of the cylinder and joint angle displacements of the boom, stick, and bucket were checked in each experiment so that it was possible to know how well the end-point of the bucket followed the reference trajectory. A comparison between the results of the collected data and the reference bucket trajectories is shown in Figure 14. Figure 14A–C shows that the performance of the draw-wire sensor is better than the IMU sensor. By inputting the data from the draw-wire and IMU sensors, the result shows that the draw-wire sensor tracked the reference better than the IMU sensor. Table 4 includes the numerical evaluation with average error (cm).

**Figure 15:**
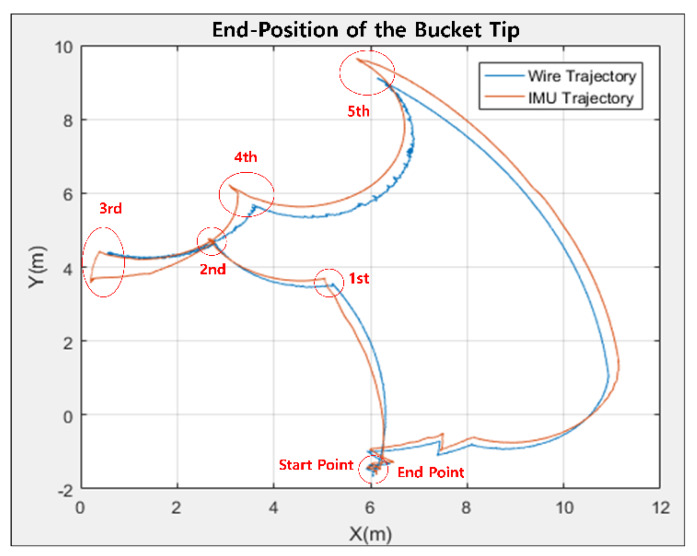
Bucket tip trajectories when all joints are moving simultaneously.

In this case of the simultaneous motion, the draw-wire sensor showed better performance than the IMU device. The area computed by the IMU data was larger than the draw-wire sensor. Figure 15 includes the resulting graph and Table 4 presents the numerical evaluation. In other words, all results illustrated that the performance of the low-cost draw-wire sensor is more accurate than the low-cost IMU device. Although the anti-vibration rubber pad was used for gathering data with little error, the results also show that the MEMS device still has weaknesses in vibrations due to the magnetic materials of the heavy-duty excavator.

## 5. Conclusions

We studied the characteristics of the position recognition of the bucket tip according to the motion measurement of excavator boom, stick, and bucket in the excavator guidance and automation. The measurement methods were divided into two ways: one is about the cylinder length and the other is of measurements of the boom, stick, and bucket links rotation. For the characteristics of each method, the wire sensor was selected for measuring the cylinder length, and the IMU sensor was selected for the measurement of the rotation of the boom, stick, and bucket link. The hardware environment was constructed to apply the selected sensor to the experimental excavator and the CAD model was also made. The accuracy of the CAD model was verified by using a protractor and image method. Then, the method to recognize the bucket tip was devised, which is available in inputting the measured data from the sensors into the excavator model. The results of recognition of the bucket tip were analyzed by experiments: first when the boom, stick, and bucket were rotated independently, and second when all of them moved simultaneously for creating a closed trajectory.

Experiments using wire sensors in a single rotation confirmed that the tip of the bucket followed the reference trajectory within 1 cm on both the x and y axes. In addition, the data measured by the wire sensor show that there is almost no change in the error of the trajectory created by the bucket tip during repeated operation. In contrast, the experiments with the IMU sensor showed that the error variance of the trajectory created by a bucket tip was larger than the draw-wire sensor. In simultaneous excavator operation, the IMU sensor measured a larger area of the closed trajectory than the wire sensor. At the third point in Figure 15, a time delay was observed to correct the accumulated error value of the IMU sensor. When measuring the cylinder length, the bucket tip trajectory was hardly affected by the disturbance caused by the movement and vibration of the excavator itself. The cylinder lengths of the boom, stick, and bucket were all measured independently by the sensor, and the independent data had almost no cumulative error compared to the IMU sensor. On the other hand, the method of measuring the rotation of the boom, stick, and bucket links was evaluated to be vulnerable to disturbance of the excavator’s own movement and vibration because the sensor data are measured based on the Earth’s gravity. Since the data of boom, stick, and bucket are all dependent, the bucket tip can be recognized with a cumulative error.

However, the draw-wire sensor has weaknesses in the aspect of installation and robustness in the hardware itself. The construction field is not in clean environments. Many debris and fragments exist so that these things can touch the wire during operations. Thus, housing satisfying desired specifications should be considered to prevent the aforementioned problems. In contrast, IMU is very simple to attach and use in the excavator. Additionally, low-cost IMU is much cheaper than draw-wire sensors. Most importantly, IMU is less affected by physical elements.

Consequently, it was verified that the cylinder length measurement method is more stable against the effects of a disturbance than the method using the IMU sensor. However, draw-wire type also has weakness in practical use. This paper focuses on the two low-cost sensor devices, which are detachable and can preserve the original status of the excavator without damage due to the mechanical modifications. This paper gives someone guidelines on what kinds of sensors would be better in machine guidance or controlling an excavator according to given external environments. 

## Figures and Tables

**Table 1:** Recent studies about sensors application in the excavator.

	Cylinder Length	Link Motion	Revolute Joint	Hydraulic	Other
		(Indirect Joint Angle)	(Direct)	Parameters	
Guidance	[3]-Magnetic	[4]-Inclination	[3]-Rotary	[5]-Hydraulic	[6]-RTLS
and	[4]-Stroke	[7]-Angle sensor	[4]-Tilt	motor	[8]-Vision
Control	[9]-Magnetic	[10]-Accelerometer	[11]-Potentiometer	[12]-Pressure	
	[13]-Draw wire	[14]-IMU	[15]-Rotary encoder		
	[16]-Draw wire	[17,18]-IMU	[19]-Potentiometer		
	[20]-Stroke	[21]-IMU	[22]-Potentiometer		
	[23]-Stroke	[24]-IMU			
		[25]-IMU			
		[26]-IMU			
		[27]-IMU			
Planning	[28]-Stroke	[29]-Magnetic			[30]-Laser
	(LVDT)				Scanner
					[31]-Vision
Other	[32]-Stroke	[33]-IMU		[34]-Pressure	[35]-Vision
	[36]-Stroke	[37]-IMU			
	[38]-magnetic				

**Table 2:** Denavit–Hartenberg table of Figure 2.

i	$\alpha_{i-1}$	$a_{i-1}$	$d_{i}$	$\theta_{i}$
1	0	0	0	$\theta_{1}$
2	0	$L_{1}$	0	$\theta_{2}$
3	0	$L_{2}$	0	$\theta_{3}$
4	0	$L_{3}$	0	0

**Table 3:** Comparison between the CAD model and real excavator in measured length.

	Length (mm)
	**Real Measurement**	**Model Gauge**	**Err**
Diagonal length (A)	2029	2041	12
Horizontal length (B)	1979	1991	12
Height (C)	447	448	1

**Table 4:** Numerical evaluation when all joints are moving separately or simultaneously.

	Average Err (cm)				Closed-Trajectory (m^2^)
**Sensor**	**Axis**	**Boom**	**Stick**	**Bucket**	**Trajectory Area**
Draw-wire	x axis	0.6	0.2	0.6	38.71
Sensor	y axis	0.9	0.9	0.9	
IMU	x axis	5.1	7.0	5.1	43.995
Sensor	y axis	7.5	6.0	7.5	

## Abbreviations

All nomenclature in this paper is as follows:

$\theta_{1}$, $\theta_{2}$, and $\theta_{3}$	link angles for foreword kinematics
$L_{1}$, $L_{2}$, and $L_{3}$	link lengths for foreword kinematics
$P_{x}$	Bucket Tip of x-axis position
$P_{y}$	Bucket Tip of x-axis position
$s_{1}$	sin($\theta_{1}$)
$c_{1}$	cos($\theta_{1}$)
$s_{12}$	sin($\theta_{1}$ + $\theta_{2}$)
$c_{12}$	cos($\theta_{1}$ + $\theta_{2}$)
$s_{123}$	sin($\theta_{1}$ + $\theta_{2}$ + $\theta_{3}$)
$c_{123}$	cos($\theta_{1}$ + $\theta_{2}$ + $\theta_{3}$)
$l_{1}$ and $l_{2}$	link lengths for boom cylinder kinematics
$l_{3}$ and $l_{4}$	link lengths for arm cylinder kinematics
$l_{5}$, $l_{6}$, $l_{7}$, $l_{8}$, and $l_{9}$	link lengths for Bucket cylinder kinematics
$l_{b}$	boom cylinder length
$l_{a}$	stick cylinder length
$l_{b}k$	bucket cylinder length
$\alpha_{b}$	boom angle by cylinder input.
